# Isolated Soy Protein Supplementation Combined With Resistance Training Improves Muscle Strength, Mass, and Physical Performance of Aging Female Mice

**DOI:** 10.3389/fphys.2022.893352

**Published:** 2022-05-26

**Authors:** Mon-Chien Lee, Yi-Ju Hsu, Fang-Yu Wu, Chi-Chang Huang, Hsueh-Yu Li, Wen-Chyuan Chen

**Affiliations:** ^1^ Graduate Institute of Sports Science, National Taiwan Sport University, Taoyuan, Taiwan; ^2^ Department of Otorhinolaryngology-Head and Neck Surgery, Sleep Center, Linkou-Chang Gung Memorial Hospital, Taoyuan, Taiwan; ^3^ Center for General Education, Chang Gung University of Science and Technology, Taoyuan, Taiwan

**Keywords:** isolated soy protein, aging, resistance training, muscle, exercise

## Abstract

**Background/Purpose:** In recent years, the aging population has gradually increased, and the aging process is accompanied by health-associated problems, such as loss of muscle mass and weakness. Therefore, it is important to explore alternative strategies for improving the health status and physical fitness of the aged population. In this study, we investigated the effect of soy protein supplementation combined with resistance training on changes in the muscle mass, muscle strength, and functional activity performance of aging mice.

**Methods:** Female Institute of Cancer Research (ICR) mice were divided into four groups (*n* = 8 per group): sedentary control (SC), isolated soy protein (ISP) supplementation, resistance training (RT), and a combination of ISP and RT (ISP + RT). The mice in designated groups received oral ISP supplementation (0.123 g/kg/day), RT (5 days/week for a period of 4 weeks), or a combination of both ISP plus RT for 4 weeks. Afterward, we assessed muscle strength, endurance, and anaerobic endurance performance and analyzed blood biochemical and pathological tissue sections to investigate whether there were adverse effects or not in mice.

**Results:** ISP supplementation effectively improved the muscle mass, muscle endurance, and endurance performance of aging female mice. The RT group not only showed similar results with ISP but also increased muscle strength and glycogen content. Nevertheless, the combination of ISP supplementation and RT had greater beneficial effects on muscle strength, physical performance, and glycogen levels (*p* < 0.05). In addition, the combination of ISP supplementation and RT had significantly increased type II muscle percentage and cross-sectional area (*p* < 0.05).

**Conclusion:** Although ISP or RT alone improved muscle mass and performance, the combination of ISP with RT showed greater beneficial effects in aging mice. Our findings suggest that regular exercise along with protein supplementation could be an effective strategy to improve overall health and physical fitness among the elderly.

## Introduction

The significant increase in life expectancy in the past 60 years is due to the decline in mortality among people in their 60s and 70s (Davis and Bailey, 1997). According to United Nations estimates, by 2050, one in six people in the world will be 65 years of age or older (He et al., 2016). The increase in life expectancy is itself a positive human development. However, aging is related to a variety of adverse reactions, leading to a decline in the ability to live independently, and many people are living with poor health and impaired physical fitness. Such changes in the elderly make them susceptible to age-related diseases, such as weakness, sarcopenia, cardiovascular disease, cancer, neurodegenerative diseases, and metabolic disorders (United Nations, 2020). Skeletal muscle is not only the largest organ of the human body but also directly affects a person’s bodily functions, is the largest amino acid library, providing essential amino acids for other key tissues/organs to synthesize new proteins for various purposes, and has the most glycogen storage (Franceschi et al., 2018). With age, skeletal muscles will undergo structural and functional changes. The reduction in muscle mass, function, fiber number, and fiber cross-sectional area may be affected by the loss of protein homeostasis, mitochondrial dysfunction, and changes in cell-to-cell communication (Jang et al., 2021), especially the pathways related to inflammation, protein turnover, and mitochondrial function (Larsson et al., 2019). In addition, muscle mass is controlled by a complex balance of muscle protein synthesis and muscle protein degradation. The ability of aging skeletal muscle to stimulate muscle protein synthesis in response to anabolic stimulation is weakened, mainly due to the impaired activation of the PI3K/Akt/mTOR/p70S6K signal axis or PI3K-Akt pathway, which is called anabolic resistance (Phillips et al., 2009; Lenk et al., 2010). The gradual decrease in muscle mass will lead to muscle atrophy. In addition to causing dysfunction, falls, and fractures, it will also increase the risk of cancer and metabolic-related diseases, such as insulin resistance, diabetes, and obesity (Fry et al., 2011). Although there is no clear treatment mechanism to improve the muscle loss caused by aging, finding alternative strategies to slow or prevent the aging-induced sarcopenia is important for the elderly.

Exercise is an effective therapy for accelerating the production of various cytokines and growth factors or regulating homeostasis and can maintain the functions of the elderly (Dalle and Koppo, 2021). Many studies demonstrated exercise as one of the strategies to improve muscle strength, muscle quality, and skeletal muscle dysfunction (Demontis et al., 2013). In addition, regular exercise helps maintain muscle mass and reduces susceptibility to age-related chronic diseases and cancer (Consitt et al., 2019). Among them, resistance training (a gradually overloaded strength training exercise in which the muscles are loaded from the outside) (Cartee et al., 2016) has been shown to have a positive effect on functional improvement and disease prevention, including muscle growth, body composition, body function, the elderly, and chronic metabolism (American College of Sports Medicine et al., 2009). Theoretically, the amount of training performed in the RT round (determined here by the formula: number of repetitions/×/group) plays an important role in chronic muscle adaptation (such as muscle size and strength) (Westcott, 2012). Compared with single-group training, acute studies have shown that multi-group training can enhance the phosphorylation of p70S6 kinase and muscle protein synthesis (Dankel et al., 2017). However, resistance exercise stimulates the rate of muscle protein breakdown to a lesser degree but stimulates the rate of muscle protein synthesis to a greater degree. When performing resistance exercise before ingesting protein, the two stimuli will synergistically combine to make the stimulation rate of muscle protein synthesis exceed the rate of muscle protein breakdown. Therefore, when combined with protein intake, repeated resistance exercises can lead to an increase in skeletal muscle protein (Terzis et al., 2010).

In addition to resistance exercise training, diet, a modifiable lifestyle factor, plays an important role in the prevention and treatment of sarcopenia (Stokes et al., 2018). Nutritional supplements, including protein omega-3, and vitamin D are considered to alleviate age-associated physical impairments and health issues (Fry et al., 2011). At present, more protein intake is considered to help in improving muscle mass and strength (Cruz-Jentoft et al., 2020). Protein supplements are rich in branched-chain amino acids (BCAAs), which can change the net balance of protein metabolism from catabolism. In addition, BCAAs composed of leucine, isoleucine, and valine have been shown to increase the level of protein synthesis and metabolism, and the synthesis of skeletal muscle protein (Jackman et al., 2017). Therefore, compared with resistance exercise alone, the combination of BCAA intake with resistance exercise can induce a higher protein synthesis rate of muscle myofibrils (Phillips and Martinson, 2019). Previous studies have shown that essential amino acids, in middle-aged mice, mainly increase mitochondrial biosynthesis and muscle function through their BCAAs and help to improve the stability of the neuromuscular junction to change nerve passage which influence muscle strength (Negro et al., 2008; D'Antona et al., 2010). In addition, it was confirmed in a long-term study that the combined treatment of RT and essential amino acids improved human muscle mass and muscle strength (Manini and Clark, 2012). Because of the potential health benefits of vegetarian and vegan diets, clinical and consumer markets are increasingly interested in them (Willoughby et al., 2007) However, plant protein is of lower quality than animal protein, so vegetarians need to consume more protein than non-vegetarians to meet their biological requirements for essential amino acids (IAAs), especially vegetarian elderly who need some high-quality protein sources (Domić et al., 2022). Soy protein has always been the preferred plant protein because it has almost complete essential amino acids. In addition, the BCAA content in the isolated soy protein (ISP) accounts for about 35% of the amino acids required for skeletal muscle formation (Melina et al., 2016) and contains isoflavones, a type of phytoestrogen that past research has shown to benefit muscle and bone in older women (Brandi et al., 1993). In addition, a higher intake of total dietary protein may overcome the different characteristics of animal protein and plant protein and their effect on muscle results (Shimomura et al., 2006).

In our previous research, we have shown that ISP combined with high-intensity interval training (HIIT) had no significant effect on the grip strength, endurance performance, and muscle mass of ovariectomized mice that simulate menopausal women, but could effectively increase bone strength and attenuate exercise-induced fatigue (Lin et al., 2018). Another study conducted on postpartum mice showed that ISP combined with HITT increased lean muscle mass, prevented weight/fat gain, improved grip and endurance performance, and promoted fatty acid oxidation in brown adipose tissue (Wei et al., 2019). However, the combined effect of ISP and resistance training on fitness variables has not yet been investigated. In this study, we aimed to explore the effect of ISP supplementation and resistance exercise training on muscle mass, strength, exercise performance, and physical fitness of 19-month-old aging female mice. We further performed histological and immunohistochemistry analyses to identify the tissue architectural changes and muscle fiber types of aging mice after the intervention.

## Materials and Methods

### Animal Care and Study Design

Female ICR mice were purchased from BioLASCO (Charles River Licensee Corp., Yi-Lan, Taiwan) and bred until 19 months of age. All mice were housed in the animal facility of the Graduate Institute of Sport Science at National Taiwan Sport University, and maintained under a stable photoperiod, temperature, and humidity conditions (12-h light/12-h dark cycle, 22 ± 2°C, and 60%–70%, respectively). During the experiment, we were provided with a standard laboratory diet (No. 5001; PMI Nutrition International, Brentwood, MO, United States) and water *ad libitum*. The Institutional Animal Care and Use Committee (IACUC) of National Taiwan Sport University and IACUC ethics committee (IACUC no. 10720) approved the animal experimentation and procedures. Thirty-two aging female mice were randomly divided into four groups (8 mice/group) for ISP supplementation and/or resistance training (RT) as follows: 1) sedentary control with vehicle (SC), 2) sedentary control with ISP supplementation (SC + ISP, 0.123 g/kg/mice/day), 3) resistance training with vehicle (RT), and 4) resistance training with ISP supplementation (RT + ISP, 0.123 g/kg/mice/day). All groups were administered with the same volume of distilled water or ISP by oral gavage. Water consumption, food intake, and animal weights were recorded twice a week.

### Isolated Soy Protein

The isolated soy protein (ISP) was purchased from Bestjet Biotechnology Co. Ltd. (New Taipei City, Taiwan). The nutrients and amino acids present in the ISP were analyzed by SGS Taiwan, Ltd. (New Taipei City, Taiwan). The nutritional information of the ISP, including hydrolyzed amino acid profiles and total branched-chain amino acids (BCAAs), is shown in Table 1.

**TABLE 1:** Nutrients, hydrolyzed amino acid profiles, and total branched-chain amino acids (BCAAs).

Nutrition facts	/100 g ISP	/100 g chow 5001
Total calories	373.6 kcal	336 kcal
Protein	83.4	23.9
Fat	3.6	5
Saturated fat	0.91	1.56
Trans fat	0	0
Carbohydrate	1.9	48.7
Sugar	0	0
Sodium	746 mg	400 mg
Hydrolyzed amino acid profiles	g/100 g ISP	/100 g chow 5001
Leucine	6.61	1.83
Valine	4.29	1.17
Isoleucine	4.26	1.14
Cystine	0.72	0.31
Tryptophan	1.07	0.29
Methionine	1.21	0.67
Threonine	3.03	0.91
Histidine	2.35	0.57
Tyrosine	2.91	0.71
Alanine	3.42	1.43
Glycine	3.44	1.21
Serine	4.15	1.19
Proline	4.46	1.49
Phenylalanine	4.62	1.04
Lysine	5.21	1.41
Arginine	6.24	1.41
Aspartic Acid	9.94	2.81
Glutamic Acid	16.99	4.37

### Resistance Training and Anaerobic Exercise Capacity Test

**FIGURE 1:**
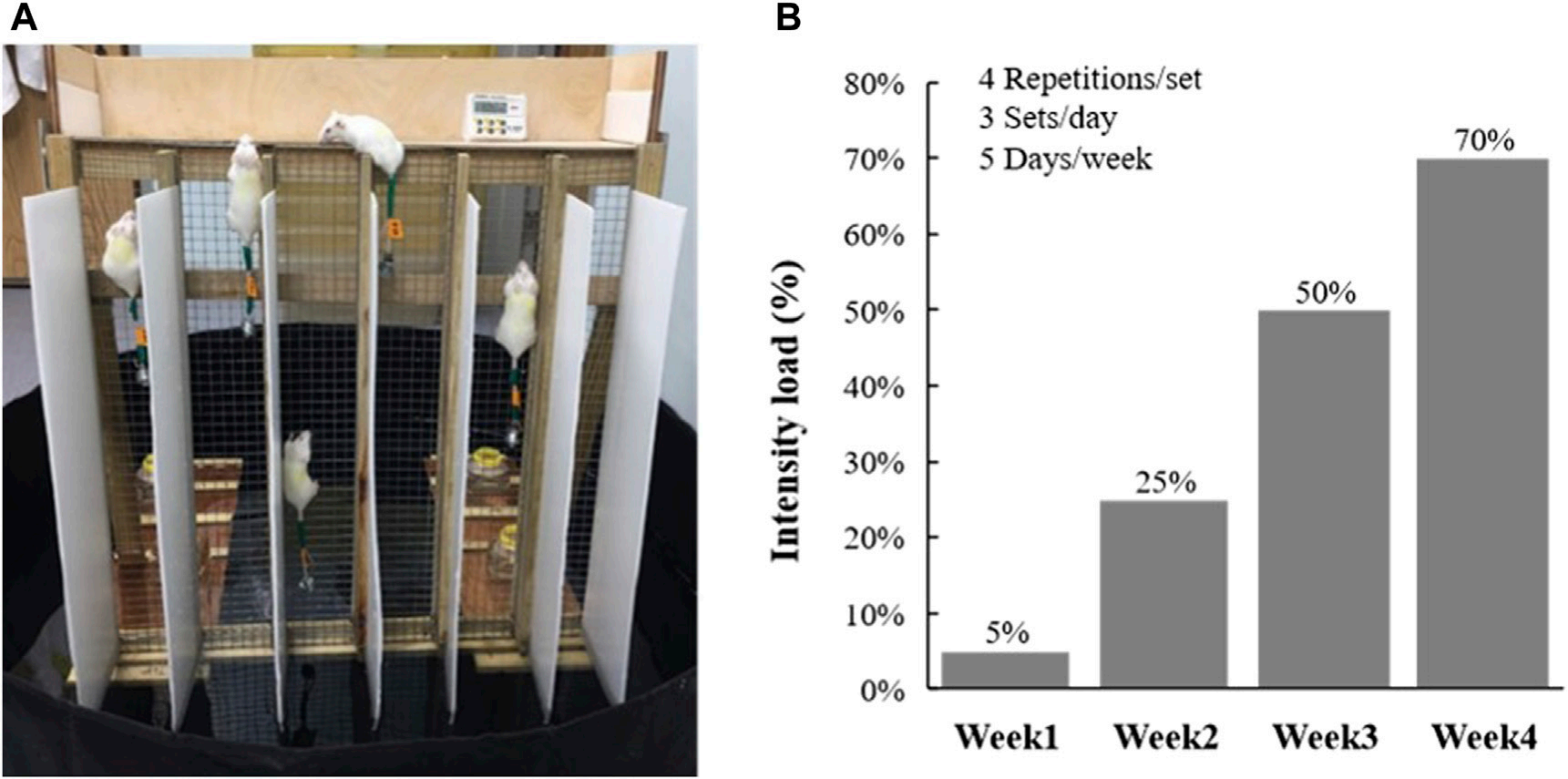
**(A)** Climbing device for resistance training. **(B)** Incremental loading intensity applied to current training protocol.

The resistance training protocol was performed 5 days/week for a period of 4 weeks. The equipment was set in water (5 cm depth) to provide negative stimulation to motivate climbing (Figure 1A) and the indicated intensity load was adjusted by individual animal weight using the protocol, as shown in Figure 1B. In resistance training, the climbing procedures were performed with four repetitions/set and three sets/day, with 1 min of rest provided between the sets (Kan et al., 2018). Performance was evaluated as the climbing time, and the number of climbs until exhaustion was used to evaluate the anaerobic performance. The criterion for exhaustion is that the mouse stagnates three times in a single crawl, and each stagnation does not climb up after five nudges or taps.

### Forelimb Grip Strength

A low-force testing system (Model-RX-5, Aikoh Engineering, Nagoya, Japan) was used to measure the grip strength of the forelimb as described previously (Lee et al., 2021).

### Endurance Exercise Performance Test

We used a motor-driven treadmill for rodents (model MK-680, Muromachi Kikai, Tokyo, Japan) to evaluate the aerobic endurance performance, and an electric shock grid was used to increase test motivation through veterinary monitoring. Before the exhaustive exercise test, all mice were initially adapted to run on a motorized treadmill at 10 m/min, 5% grade, for 5 min/day for a week. On the test, we set a fixed slope of grade 15° and an initial speed of 15 m/min for mice running on the treadmill, then, every subsequent 2 min, the speed was increased by 3 m/min until the mice maintained continuous contact with the shock grid for 5 s, that we defined it to exhaustion (Kan et al., 2018).

### Clinical Biochemical Profiles

A series of blood biochemical assessments were performed to evaluate the functional ability of essential body organs. At the end of the experiments, all mice were sacrificed by 95% CO_2_ asphyxiation, and the blood sample was collected immediately at rest. Serum was collected by centrifugation, and the levels of alanine aminotransferase (ALT), aspartate aminotransferase (AST), albumin (ALB), total cholesterol (TC), triacylglycerol (TG), blood urea nitrogen (BUN), creatinine (CREA), uric acid (UA), total protein (TP), creatine kinase (CK), and lactate dehydrogenase (LDH) were assessed by an auto-analyzer (Hitachi 717, Hitachi, Tokyo, Japan).

### Body Composition and Glycogen Content Analysis

After the mice were euthanized, the important body organs, including the liver, kidney, heart, lung, muscle (gastrocnemius), MT (thigh muscle), ovary fat pad (OFP), and brown adipocyte tissue (BAT), were accurately excised and weighed. Among them, the muscle and liver tissues were kept at −80°C for a subsequent glycogen content analysis. For the assay, 100 μg of liver and muscle tissue were homogenized in 500 μl cold perchloric acid and then centrifuged at 15,000 × g for 15 min at 4°C. The resultant supernatant was collected before determining the glycogen concentration. We used a commercial assay kit (Sigma-Aldrich, St. Louis, MO, United States) according to the manufacturer’s instructions to determine the levels of glycogen in the liver and muscle (mg/g).

### Histopathological and Immunohistochemical Staining

The liver, kidney, muscle, MT, heart, lung, OFP, and BAT tissues were fixed in 10% formalin, embedded in paraffin, and cut into 4 μm-thick sections for morphological and pathological evaluation. Tissue sections were stained with hematoxylin and eosin (H & E) and examined by light microscopy with a CCD camera (BX-51, Olympus, Tokyo, Japan) by a clinical pathologist.

The muscle tissues (gastrocnemius) were further analyzed to see the effects of training and ISP supplementation on type I and type II fiber types. Primary antibodies of myosin-heavy chain fast (WB-MHCf) and myosin-heavy chain slow (WB-MHCs) were purchased from Novocastra (Leica Biosystem, Wetzlar, Germany) and applied to distinguish the fiber types. ER2 repair solution (AR9640, Leica Biosystem, Wetzlar, Germany) was used to repair the epitopes of MHCf and MHC, and then performed the initial incubation. The detection kits (Bond Polymer Refine Detection & Bond Polymer Refine Red Detection) used an automated BondMax double staining system, as previously described (Kan et al., 2018). The cross-sectional area (CSA, μm^2^) of a muscle, and type I muscle observed per high-power-field (HPF), were measured and analyzed using ImageJ software (NIH, MD, United States).

### Statistical Analysis

Data were expressed as mean ± SD. Statistical analyses were performed using SAS v9.0 (SAS, Cary, NC, United States). Two-way ANOVA was performed to assess the effect of exercise training and ISP supplementation on all the experimental data. *p* < 0.05 was considered statistically significant.

## Results

### General Characteristics of Aging Mice With ISP Supplementation and RT

As shown in Table 2, the body weights of mice were not significantly changed after 4 weeks of ISP supplementation or in combination with RT. However, water (*p* < 0.0001) and diet (*p* < 0.0001) intake were significantly lower in ISP and ISP plus RT groups compared with SC and RT groups (Table 2).

**TABLE 2:** General characteristics of the experimental groups.

Characteristics	SC	ISP	RT	RT + ISP	Main factor *p* value		
					ISP	RT	RT + ISP
Initial BW (g)	42.4 ± 2.80^a^	42.2 ± 3.50^a^	42.2 ± 4.50^a^	42.6 ± 4.10^a^	0.8406	0.9483	0.8140
Final BW (g)	43.4 ± 2.70^a^	42.7 ± 3.70^a^	42.7 ± 4.60^a^	43.1 ± 4.10^a^	0.3338	0.0879	0.7832
Food intake (g/day)	6.24 ± 0.80 ^b^	5.19 ± 1.51^a^	6.10 ± 0.86 ^b^	5.21 ± 1.14^a^	<0.0001	0.3003	0.6840
Water intake (ml/day)	11.18 ± 1.42^c^	10.15 ± 1.54^b^	11.78 ± 1.75^c^	9.05 ± 1.42^a^	<0.0001	0.6840	0.6628
Liver (g)	2.14 ± 0.31^a^	2.12 ± 0.29^a^	2.18 ± 0.18^a^	2.12 ± 0.34^a^	0.6880	0.8479	0.8479
Kidney (g)	0.67 ± 0.12^a^	0.69 ± 0.13^a^	0.64 ± 0.09^a^	0.65 ± 0.13^a^	0.3993	0.7324	0.9881
OFP (g)	0.35 ± 0.12^a^	0.34 ± 0.12^a^	0.34 ± 0.10^a^	0.34 ± 0.08^a^	0.8687	0.7164	0.9473
Heart (g)	0.23 ± 0.02^a^	0.24 ± 0.03^a^	0.23 ± 0.01^a^	0.24 ± 0.02^a^	0.1865	0.8129	0.8129
Lung (g)	0.32 ± 0.04^a^	0.32 ± 0.05^a^	0.33 ± 0.06^a^	0.33 ± 0.04^a^	0.7234	0.5252	1.0000
Muscle (g)	0.26 ± 0.02^a^	0.30 ± 0.03 ^b^	0.30 ± 0.02 ^b^	0.30 ± 0.03 ^b^	0.0745	0.0424	0.0973
MT (g)	0.41 ± 0.05^a^	0.41 ± 0.06^a^	0.41 ± 0.03^a^	0.41 ± 0.03^a^	0.9691	0.9691	0.9691
BAT (g)	0.10 ± 0.02^a^	0.09 ± 0.03^a^	0.09 ± 0.02^a^	0.11 ± 0.02^a^	0.9421	0.6122	0.1353
Relative liver weight (%)	5.11 ± 0.70^a^	5.26 ± 1.24^a^	5.48 ± 0.54^a^	5.53 ± 1.33^a^	0.7808	0.3782	0.8941
Relative kidney weight (%)	1.61 ± 0.29^a^	1.70 ± 0.43^a^	1.59 ± 0.14^a^	1.68 ± 0.31^a^	0.4244	0.8529	0.9955
Relative OFP weight (%)	0.83 ± 0.26^a^	0.84 ± 0.32^a^	0.85 ± 0.26^a^	0.87 ± 0.24^a^	0.8820	0.8516	0.9434
Relative heart weight (%)	0.55 ± 0.06^a^	0.58 ± 0.09^a^	0.57 ± 0.04^a^	0.62 ± 0.10^a^	0.1365	0.3518	0.7887
Relative lung weight (%)	0.76 ± 0.10^a^	0.80 ± 0.21^a^	0.82 ± 0.13^a^	0.87 ± 0.14^a^	0.3811	0.2515	0.9815
Relative muscle weight (%)	0.61 ± 0.06^a^	0.70 ± 0.09 ^b^	0.70 ± 0.05 ^b^	0.70 ± 0.07 ^b^	0.1037	0.0629	0.0772
Relative MT weight (%)	0.98 ± 0.14^a^	1.02 ± 0.26^a^	1.03 ± 0.11^a^	1.06 ± 0.17^a^	0.5466	0.4724	0.9378
Relative BAT weight (%)	0.24 ± 0.06^a^	0.22 ± 0.10^a^	0.23 ± 0.05^a^	0.28 ± 0.08^a^	0.6338	0.3933	0.2574

Data are expressed as mean ± SD for n = 10 mice in each group. (1) Sedentary control with vehicle (SC), (2) sedentary control with ISP supplementation (SC + ISP, 0.123 g/kg/mice/day), (3) resistance training with vehicle (RT), and (4) resistance training with ISP supplementation (RT + ISP, 0.123 g/kg/mice/day). Data in the same row with different letters (a, b) differ significantly at *p* < 0.05 by two-way ANOVA; OFP: ovary fat pad; BAT: brown adipose tissue.

On the body composition, no significant differences were observed in absolute or relative weights of the liver, kidney, heart, lung, MT, OFP and BAT tissues among the groups. However, muscle weights in ISP, RT, and ISP plus RT groups were significantly greater than in SC by 1.13-fold (*p* = 0.0112), 1.12-fold (*p* = 0.0176), and 1.14-fold (*p* = 0.0089), respectively, and only supplementation had a significant effect (*p* = 0.0424). Similarly, relative muscle weights in ISP, RT, and ISP plus RT groups were significantly greater than in SC by 1.15-fold (*p* = 0.0126), 1.15-fold (*p* = 0.0191), and 1.15-fold (*p* = 0.0062), respectively, but had no significant main effect of exercise or ISP (Table 2).

### ISP Supplementation and RT Improves Grip Strength of Aging Mice

**FIGURE 2:**
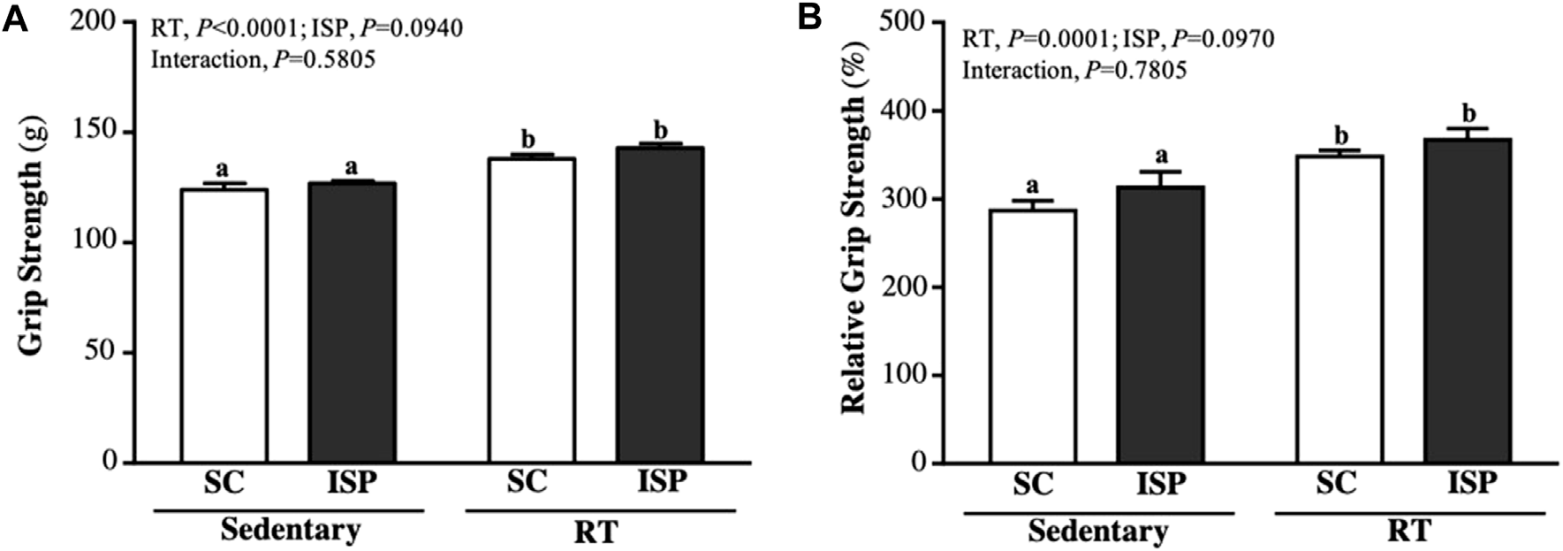
Effect of ISP supplementation and RT of aging mice on **(A)** absolute forelimb grip strength and **(B)** forelimb grip strength (%) relative to body weight. Data are expressed as mean ± SD for *n* = 8 mice per group. Different superscript letters (a, b, and c) indicate significant difference at *p* < 0.05.

The forelimb grip strength of mice in SC, ISP, RT, and ISP + RT groups were, 124 ± 8, 127 ± 4, 138 ± 5, and 143 ± 7 (g), respectively. The grip strength of mice in RT and ISP + RT groups were significantly higher than in SC by 1.11-fold (*p* = 0.0002) and 1.15-fold (*p* < 0.0001), respectively, with only exercise as the main effects (*p* < 0.0001) (Figure 2A). The relative grip strength (%), normalized to body weight was found to be significantly higher in RT and ISP + RT groups than in SC by 1.21-fold (*p* = 0.0021) and 1.28-fold (*p* = 0.0001), respectively, with only exercise as the main effects (*p* = 0.0001) (Figure 2B).

### Effect of ISP Supplementation and RT on the Anaerobic Exercise Performance of Aging Mice

**FIGURE 3:**
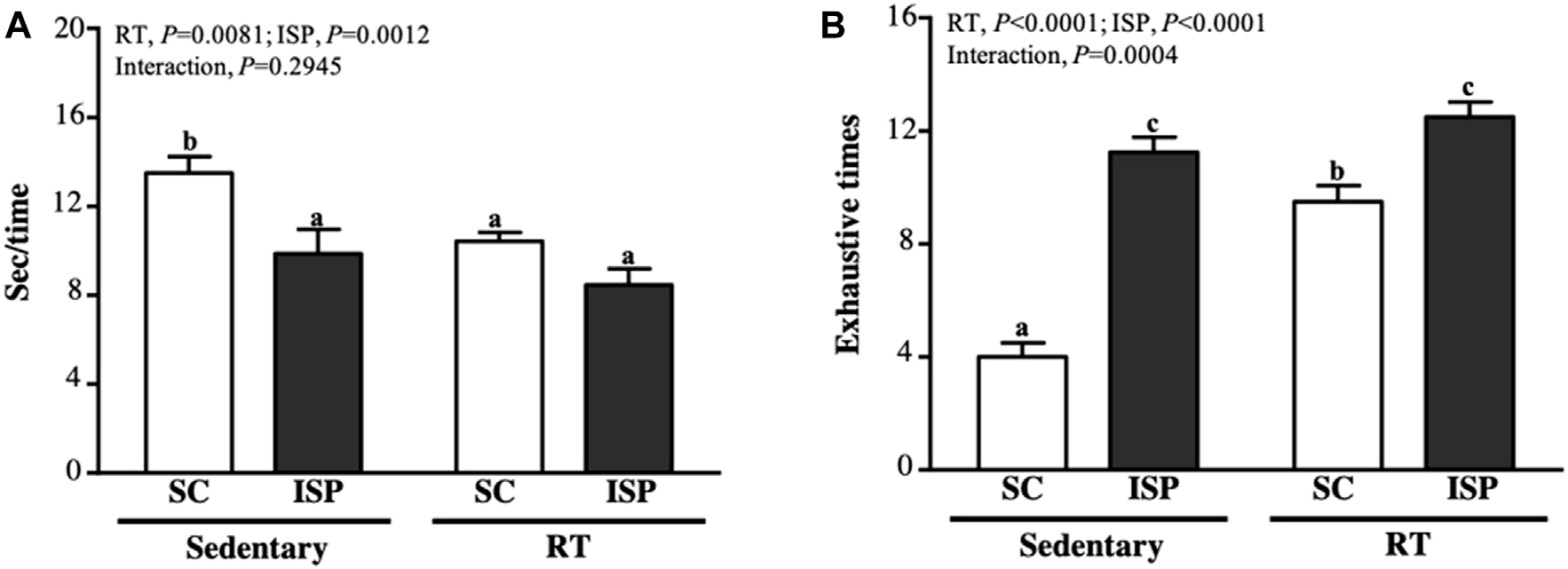
Effect of ISP supplementation and RT of aging mice on **(A)** time for each climbing and **(B)** exhaustion times for climbing. Data are expressed as mean ± SD for *n* = 8 mice per group. Different superscript letters (a, b, and c) indicate significant difference at *p* < 0.05.

**FIGURE 4:**
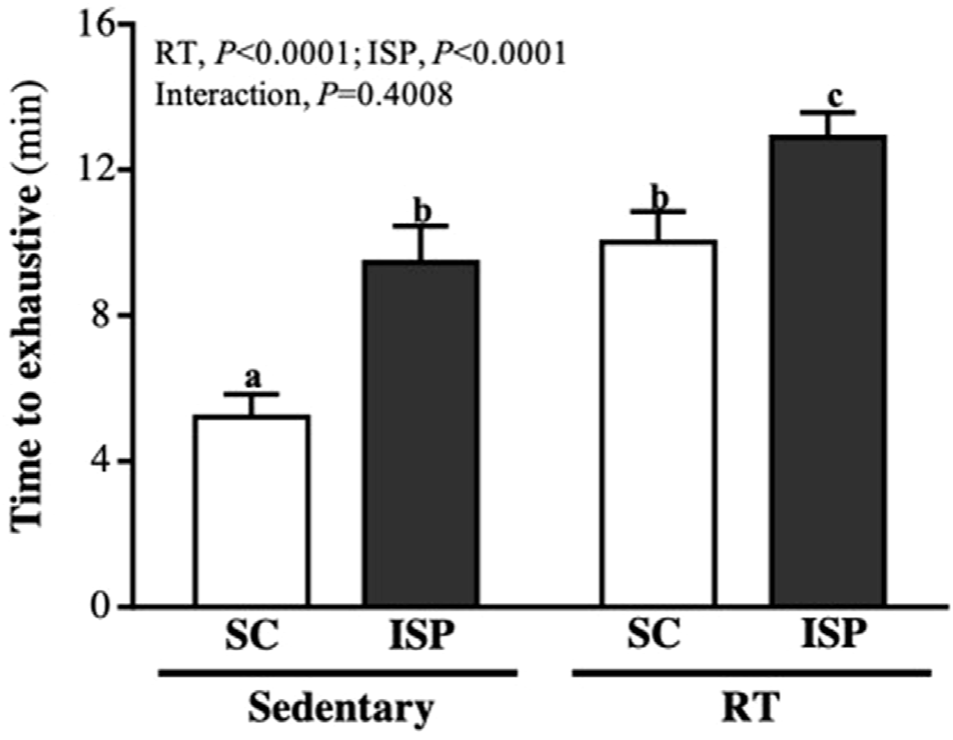
Effect of ISP supplementation and RT of aging mice on endurance exercise performance. Data are expressed as mean ± SD for *n* = 8 mice per group. Different superscript letters (a, b, and c) indicate significant difference at *p* < 0.05.

We used speed and the maximum number of repetitions to measure the anaerobic exercise performance. In terms of climbing speed, except SC mice (13.49 ± 2.13 s), all other groups (ISP, RT, and ISP + RT) spent less time (9.87 ± 3.08, 10.43 ± 1.13, and 8.47 ± 2.03 s, respectively) to complete the climbing (Figure 3A). The decreased climbing time of aging mice in ISP + RT was prominent (37.21%; *p* < 0.0001) than in ISP (26.79%; *p* = 0.0027) and RT (22.69%; *p* = 0.0098) groups. The main effect of ISP (*p* = 0.0081) and RT (*p* = 0.0012) was the significantly increased climbing speed, but there was no interaction effect (Figure 4A). The repetition maximum (RM), an index of muscular endurance performance of mice in SC, ISP, RT, and ISP + RT groups was 4.00 ± 1.41, 11.25 ± 1.49, 9.50 ± 1.60, and 12.50 ± 1.51 (times), respectively (Figure 3A). Compared with SC, the exhaustive time in ISP, RT, and ISP + RT groups was significantly longer by 2.81-fold (*p* = 0.0027), 2.38-fold (*p* = 0.0098), and 3.13-fold (*p* < 0.0001), respectively. In addition, the exhaustive time in ISP and ISP + RT groups was significantly longer than in RT group by 1.18-fold (*p* = 0.0276) and 1.32-fold (*p* = 0.0004), respectively. The main effect of ISP (*p* < 0.0001) and RT (*p* < 0.0001) was significantly increased muscular endurance performance, and had a significantly interactive effect (*p* = 0.0004) (Figure 3B).

### ISP Supplementation With RT Promotes Exercise Performance in Aging Mice

As seen in Figure 4, the time to exhaustion in SC, ISP, RT, and ISP + RT groups was 5.21 ± 1.80, 9.46 ± 2.83, 10.02 ± 2.38, and 12.90 ± 1.92 (min), respectively. The climbing time of mice in ISP, RT, and ISP + RT groups was significantly longer than the SC group by 1.81-fold (*p* = 0.0008), 1.92-fold (*p* = 0.0002), and 2.74-fold (*p* < 0.0001), respectively. Among them, the ISP + RT group represented with the most effective improvement. The main effect of ISP (*p* < 0.0001) and RT (*p* = 0.0001) was the significantly increased endurance exercise performance, but there was no significant interactive effect.

### Combination of ISP and RT Preserves Liver and Muscle Glycogen Levels in Aging Mice

**FIGURE 5:**
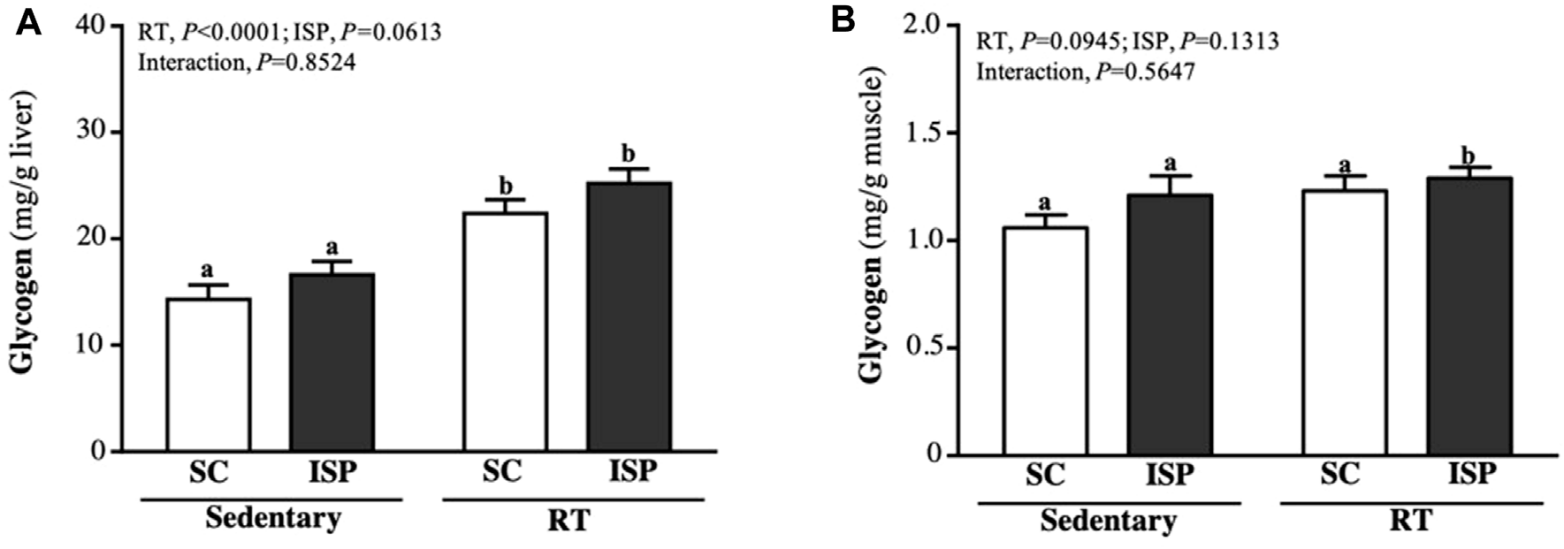
Effect of ISP supplementation and RT of aging mice on **(A)** liver glycogen and **(B)** muscle glycogen. Data are expressed as mean ± SD for *n* = 8 mice per group. Different superscript letters (a and b) indicate significant difference at *p* < 0.05.

Glycogen is mainly present in the liver and skeletal muscle and is used for energy demand and homeostasis. Aging mice liver glycogen levels were found to be higher in RT (22.38 ± 3.67 mg/g) and ISP + RT groups (25.20 ± 3.86 mg/g). Compared with SC, liver glycogen levels in RT and ISP + RT groups were significantly higher by 1.57-fold (*p* = 0.0002) and 1.76-fold (*p* < 0.0001), respectively (Figure 5A). The muscle glycogen content in SC, ISP, RT, and ISP + RT groups were 1.06 ± 0.18, 1.21 ± 0.26, 1.23 ± 0.20, and 1.29 ± 0.15 (mg/g), respectively. In contrast to the liver, higher muscle glycogen levels were maintained only in the ISP + RT group (1.22-fold; *p* = 0.0277) (Figure 5B).

### Effect of ISP Supplementation and RT on Blood Biochemical Parameters in Aging Mice

At the end of the experiment, we further performed a blood biochemical analysis to explore the effect of ISP and RT on various key clinical outcomes. In the results, we found that there were no significant differences in the levels of AST, ALT, ALB, TC, TG, CREA, UA, TP, CK, LDH, and glucose among the groups (Table 3). However, serum BUN levels were significantly increased with ISP supplementation and also with a combination of RT. The elevated BUN levels in ISP and ISP + RT groups were 1.10-fold (*p* = 0.0235) and 1.12-fold (*p* = 0.0087), respectively (Table 3).

**TABLE 3:** Effect of ISP supplementation and RT on biochemical assessments of serum at the end of the experiment.

Parameter	SC	ISP	RT	RT + ISP	Main factor *p* value		
					ISP	RT	RT + ISP
AST (U/L)	88 ± 10^a^	85 ± 9^a^	86 ± 7^a^	88 ± 7^a^	0.8972	0.9656	0.3921
ALT (U/L)	52 ± 5^a^	51 ± 9^a^	50 ± 4^a^	52 ± 9^a^	0.7884	0.7512	0.6090
TC (mg/dl)	131 ± 13^a^	124 ± 11^a^	128 ± 20^a^	129 ± 16^a^	0.5679	0.9195	0.4675
TG (mg/dl)	87 ± 17^a^	84 ± 11^a^	86 ± 13^a^	86 ± 14^a^	0.8404	0.9799	0.7626
LDH (mg/dl)	447 ± 92^a^	434 ± 119^a^	491 ± 83^a^	472 ± 65^a^	0.6289	0.2120	0.9301
ALB (g/dl)	3.09 ± 0.2^a^	3.12 ± 0.61^a^	3.18 ± 0.35^a^	3.05 ± 0.21^a^	0.7464	0.9631	0.5490
CPK (U/L)	259 ± 70^a^	273 ± 83^a^	259 ± 67^a^	268 ± 81^a^	0.6764	0.9172	0.9319
TP (g/dl)	5.18 ± 0.36^a^	5.24 ± 0.32^a^	0.38 ± 0.12^a^	5.21 ± 0.46^a^	0.6797	0.4714	0.3559
BUN (mg/dl)	21.5 ± 1.7^a^	23.7 ± 1.8 ^b^	22.4 ± 1.7^a^	24.1 ± 2.1^b^	0.0048	0.3631	0.4758
CREA (mg/dl)	0.38 ± 0.03^a^	0.38 ± 0.03^a^	0.39 ± 0.02^a^	0.39 ± 0.03^a^	0.6282	0.3710	0.9447
UA (mg/dl)	2.25 ± 1.07^a^	2.30 ± 0.68^a^	2.23 ± 0.38^a^	2.36 ± 1.05^a^	0.7720	0.9537	0.8924
Glucose (mg/dl)	189 ± 24^a^	195 ± 20^a^	186 ± 19^a^	193 ± 18^a^	0.3863	0.6853	0.9656

Data are expressed as mean ± SD for n = 10 mice in each group. (1) Sedentary control with vehicle (SC), (2) sedentary control with ISP supplementation (SC + ISP, 0.123 g/kg/mice/day), (3) resistance training with vehicle (RT), and (4) resistance training with ISP supplementation (RT + ISP, 0.123 g/kg/mice/day). Data in the same row with different letters (a, b) differ significantly at *p* < 0.05 by two-way ANOVA. AST, aspartate aminotransferase; ALT, alanine aminotransferase; TC, total cholesterol; TG, triglycerides; LDH, lactate dehydrogenase; ALB, albumin; CK, creatine kinase; TP, total protein; BUN, blood urea nitrogen; CREA, creatinine; UA, urea acid.

### Effect of ISP Supplementation and RT on the Histological Observations of Aging Mice

**FIGURE 6:**
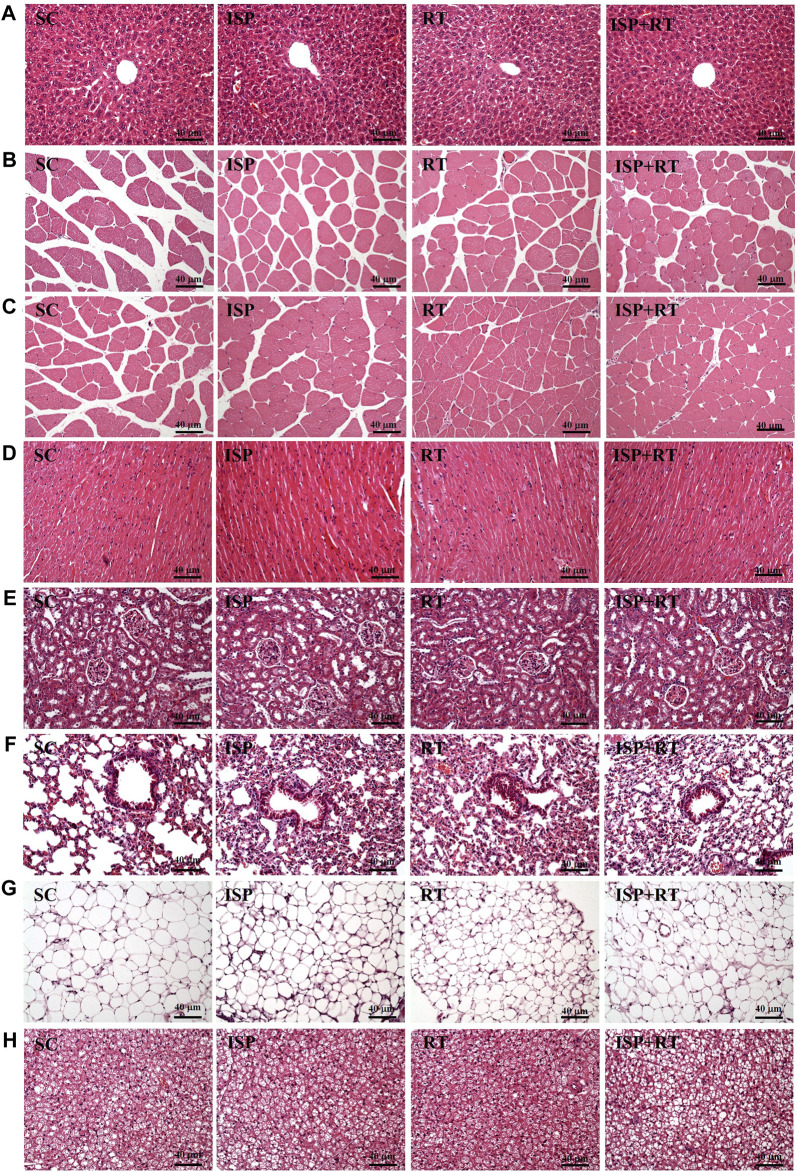
Effect of ISP supplementation and RT of aging mice on **(A)** liver, **(B)** thigh muscle, **(C)** muscles, **(D)** heart, **(E)** kidney, **(F)** lung, **(G)** OFP, and **(H)** BAT tissue in mice (H&E stain, magnification: ×200; bar, 40 μm; BAT magnification: ×100; bar, 80 μm).

At the end of the study, histological examinations of the liver, MT, muscle, heart, kidney, lung, OFP, and BAT of aging mice were performed. As shown in the images, no abnormalities were observed in aging tissues of all experimental groups (Figure 6). After the prescribed treatment, the arrangement of hepatic sinusoids and hepatic cords in the liver showed no change. Only large senile liver nucleoli appeared in each group, which is a normal senescence phenomenon. In addition, Zenker degeneration and hyperplasia were not observed in the skeletal muscle or cardiomyocytes. The structures of renal tubules and glomeruli were also not different among the treatment groups.

### Effect of ISP Supplementation and RT on the Muscle Fiber Types and Morphology of Aging Mice

**FIGURE 7:**
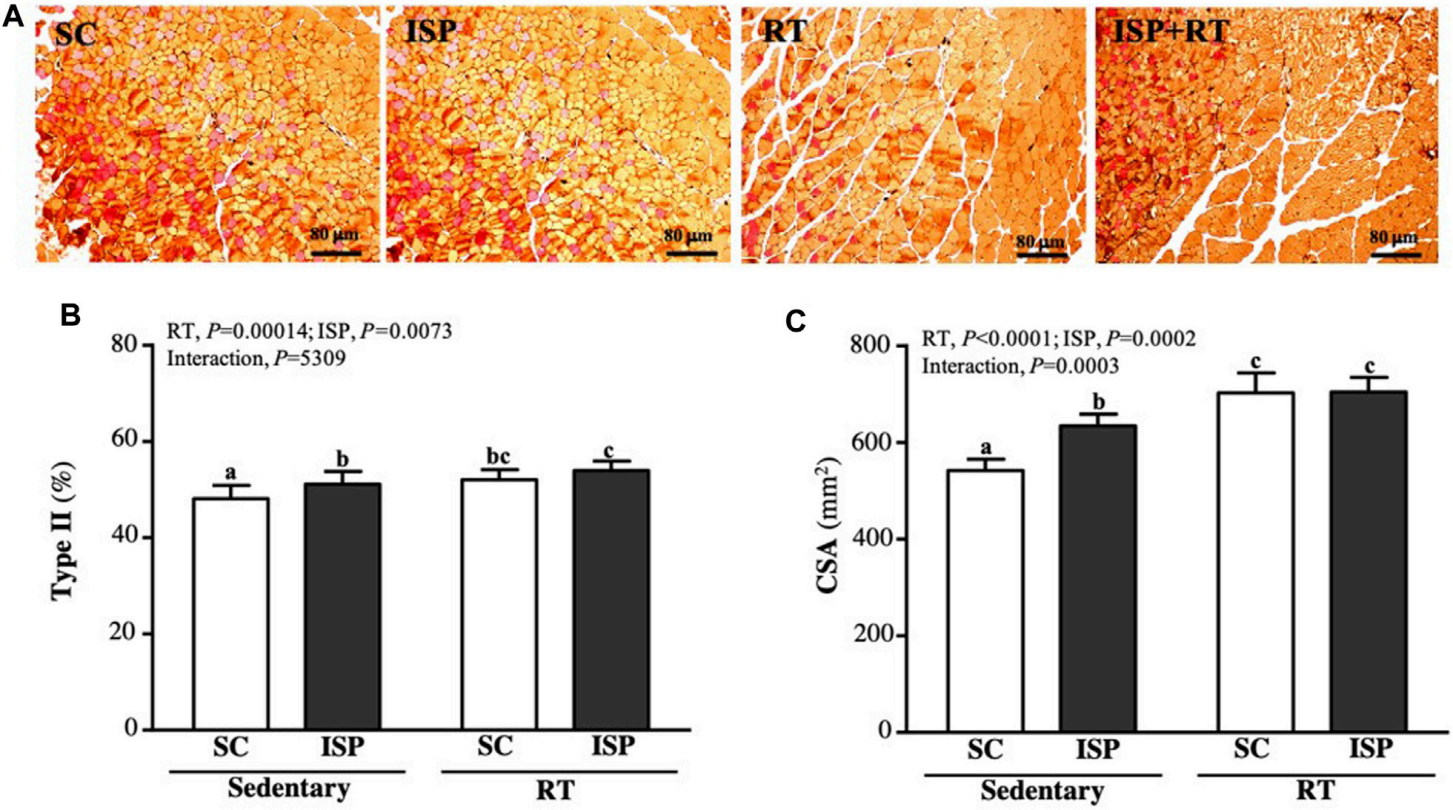
Effect of ISP supplementation and RT of aging mice on **(A)** muscle of thigh with IHC staining, **(B)** muscular type proportions, and **(C)** cross section area (CSA). Specimens were photographed under a light microscope (Hematoxylin and eosin stain, magnification: ×200; scale bar, 40 μm). Data are expressed as mean ± SD for *n* = 8 mice per group. Different superscript letters (a and b) indicate significant difference at *p* < 0.05.

We further analyzed the ratio of type I and type II fibers and the cross-sectional area (CSA) of the thigh muscles to verify the effect of resistance exercise and ISP supplementation on aging mice. Type I and type IIa fibers were reddish in color, while type II was brownish in color (Figure 7A). Figure 7B showed the percentage of type II muscle in total muscle. The type II fiber percentage in SC, ISP, RT, and ISP + RT groups were 48.13 ± 2.75, 51.12 ± 2.65, 52.05 ± 2.13, and 53.97 ± 1.98 (%), respectively. Here, we noticed that ISP, RT, and ISP + RT interventions significantly increased type II fiber percentage by 1.06-fold (*p* = 0.0188), 1.08-fold (*p* = 0.0027), and 1.12-fold (*p* < 0.0001), respectively compared with SC. The main effects were ISP (*p* = 0.0004) and RT (*p* = 0.0073), but there was no significant interactive effect. In addition, the CSA of the SC muscle was 543 ± 24 μm^2^, while the CSA of ISP, RT, and ISP + RT groups was 635 ± 24, 703 ± 41, and 705 ± 30 (μm^2^), respectively. The CSA scores in ISP, RT, and ISP + RT groups were 1.17-fold (*p* < 0.0001), 1.30-fold (*p* < 0.0001), and 1.30-fold (*p* < 0.0001), respectively, greater than in SC. The main effects of ISP (*p* < 0.0001) and RT (*p* = 0.0002) were the significantly increased percentage of type I muscle, and had a significant interactive effect (*p* = 0.0003) (Figure 7B).

## Discussion

In this study, we demonstrated the influential role of ISP supplementation alone and also in combination with resistance training on aging mice’s muscle strength, endurance performance, muscle mass, and muscle histological changes. Our findings revealed that ISP supplementation alone significantly improved the muscle mass, muscle endurance, and endurance performance of aging mice. The RT not only had similar effects as ISP, but also increased the muscle strength and liver glycogen content. Nevertheless, the combination of ISP supplementation plus RT had greater beneficial effects, and this was evidenced by improved muscle strength, glycogen storage, and physical performance in aging mice.

First, we found decreased food and water intake after ISP supplementation; however, this did not result in decreased bodyweights of the mice. We assume that ISP intake might increase the feeling of satiety in mice, and thereby decreased food intake. In this study, aging female mice were used (19-month), so the body weight might be maintained at a high but stable state. Previous studies have shown that weight gain is not directly related to muscle mass and strength. However, muscle mass is the main determinant of muscle strength (Reid et al., 2008). The muscles after exercise are more sensitive to nutrition and can synthesize more available amino acids into skeletal muscle protein (Schoufour et al., 2019). RT and essential amino acids have independent effects. Both of these interventions can stimulate muscle proteins to replace old, non-functional proteins with new functional proteins, and increase muscle protein synthesis faster than muscle protein breakdown. Therefore, under the two synergistic effects, it has the benefit of increasing net muscle protein synthesis (Tipton et al., 1999; Franceschi et al., 2018). In addition, acute exercise combined with the ingestion of protein or amino acids can enhance the muscle protein anabolic response by activating the mTORC1 pathway, which is beneficial for promoting recovery following exercise and may improve muscle mass and quality over the long term (Burd et al., 2009). However, resistance training and protein supplementation are not as effective for the elderly as for the young, which is called the chronic slow response of the elderly (Kumar et al., 2009). In most sedentary elderly subjects, the sensitivity of skeletal muscle tissue to anabolic stimulation by physical activity or protein intake may be reduced. A previous study had shown that after 14 days of reduced physical activity in the elderly, the postprandial muscle protein synthesis rate is significantly reduced by 26% (Breen et al., 2013). Nevertheless, the combination of exercise and increasing protein intake is still one of the best strategies. A previous study showed that dietary protein supplementation after resistance exercise training increased muscle protein synthesis in the elderly by 28% (Pennings et al., 2011). In another long-term trial, 24 weeks of protein supplements combined with resistance training increased the muscle mass, strength, and physical function of frail elderly participants (Tieland et al., 2012). Therefore, in this study, we used 19-month-old aging female mice and supplemented them with ISP in combination with RT for 4 weeks. Our findings showed improved muscle mass with ISP and RT alone, and also with a combination of both ISP plus RT. Although RT and ISP + RT effectively improved the maximum muscle performance, ISP, RT, and ISP + RT intervention effectively increased the muscle endurance and anaerobic exercise performance of aging mice. Furthermore, time to exhaustion (climbing) and relative grip strength was significantly higher with the combination of ISP plus RT. These findings emphasize the benefits of ISP plus RT synergistic effect on improving the muscle mass, strength, and physiology of aging mice.

As age leads to a decrease in muscle mass, the type II muscle fiber atrophy may appear in muscle fibers (Nilwik et al., 2013; McCormick and Vasilaki, 2018), accompanied by a type-specific decrease in the number and function of skeletal muscle stem cells or satellite cells (Dreyer et al., 2006). However, resistance exercise training more than three times a week has been shown as an effective strategy to increase the quality and strength of skeletal muscles in the elderly (Peterson et al., 2011). In addition, a previous study has pointed out that long-term resistance exercise training in the elderly can restore the content of type II muscle fiber satellite cells to the level of untrained healthy young people, and improve the response of acute type II muscle fiber satellite cells. In this study, we found that ISP supplementation and RT alone, and a combination of both (ISP + RT) significantly increased the percentage of type II muscle fiber types in aging mice. This was further witnessed by the increased size of the muscle fiber types CSA. A previous study demonstrated that when RT is performed, animals show significant muscle remodeling, which is characterized by a decrease in the bulk density of slow and medium-speed fibers, with a significant increase in the bulk density of fast fibers (Leenders et al., 2013). In particular, RT related to a plant protein diet can be an auxiliary factor in inhibiting or reversing the sarcopenia by promoting the slight recovery of rapid glycolytic fiber atrophy, and accompanied by an increase in collagen (Figueiredo Braggion et al., 2016).

Muscular glycogen content affects the quantity and quality of muscle fibers. In addition to stable glycogen reserves, our study further confirmed that ISP, RT, and ISP + RT effectively increased skeletal muscle mass, muscle strength, and muscle fiber CSA. It has been claimed that the skeletal muscle glucose transporter type 4 (GLUT4) protein would increase with RT, but this may depend on the body composition and metabolic status (diabetes) before training. Six weeks of single-leg strength training (three times a week) increased skeletal muscle GLUT4 (∼40%) of the exercised leg in the elderly and non-obese type 2 diabetic patients, which helps to improve the insulin sensitivity. Higher levels of glycogen in the skeletal muscle may be due to the increased insulin sensitivity (Holten et al., 2004). Another study conducted on men aged 50–63 found that resistance training for 16 weeks increased insulin-stimulated non-oxidized glucose processing (40%). This phenomenon may help in improving systemic insulin sensitivity (22%), which revealed that RT can improve skeletal muscle glycogen metabolism (Miller et al., 1994). In addition, after 6 weeks of RT, both healthy and diabetic elderly had increased skeletal muscle glycogen content (∼16%) and significantly increased basal glycogen synthase activity (∼9% and 20%, respectively) (Holten et al., 2004). Glycogen is the storage form of glucose (energy) in mammals. Most glycogen is produced and stored by liver (∼100 g) and muscle (∼350–700 g) cells, and glycogen content depends on the training status, diet, muscle fiber type composition, gender. and weight of an individual. Glucose output in the liver is the main source of glucose available to increase muscle exercise. Increased liver glycogen storage helps in maintaining the constant blood sugar and it is a key factor in improving endurance (Knuiman et al., 2015). Knuiman et al. (2015) conducted experiments on mice with high concentrations of liver glycogen to explore the correlation between energy reserves and performance. Under a low-intensity running program, these mice can run farther, indicating that liver glycogen is closely related to the endurance ability (López-Soldado et al., 2021). It is well known that glycogen is mainly derived from carbohydrate intake and converted into glucose storage. However, supplementation of BCAAs also increases glycogen storage. In a previous study, young rats were supplemented with 45 mg BCAA/body weight per day to perform 5-weight swimming training. The results showed that supplementation significantly increased the glycogen content in the liver (de Campos-Ferraz et al., 2011). Similarly, we reported that supplementation of ISP combined with RT effectively improved glycogen reserves in aging mice, and this was accompanied by improved muscle strength, muscle mass, and performance. Increased muscle fiber CSA and fiber type transformation after the combination treatment further supports the beneficial effects of ISP in aging mice. Taken together, the combination treatment promotes glycogen reserves and exercise performance in aging mice without adverse effects.

In addition to physical performance and functional testing of aging female mice supplemented with ISP and RT, we also performed blood analysis and histopathological interpretation to confirm that the health status of aging mice and interventional substances did not cause the risk of injury. In terms of histopathology, except for the appearance of aging under normal conditions, no other damages were observed. In the blood analyses, the liver function biomarkers and lipid profile did not significantly differ with any of the treatments in aging mice. For the kidney function assessments, only BUN was found to be significantly increased in the ISP supplement group, but this increase was still in the normal range. This is a normal phenomenon, because BUN is a serum by-product of protein metabolism, formed by the liver and carried by the blood to the kidneys for excretion (Wang et al., 2021). Therefore, higher protein intake will result in higher BUN concentration under normal metabolism.

In recent years, the demand for the use of plant protein has increased, and a growing number of studies have compared the effects of various plant and animal protein sources in stimulating muscle protein synthesis, improving exercise training fitness, and enhancing physique (Kerksick et al., 2021). Past studies have compared net protein utilization values using the protein digestibility corrected amino acid score (PDCAAS), a similar dichotomy. For example, on a scale of 100, plant sources range from 53–67, while animal sources range from 73–94 (Schaafsma, 2000). Nonetheless, one study noted that 48 untrained men and women were randomized over 12 weeks to either 19 g of whey protein isolate or 26 g of soy protein isolate, both containing a protein dose of 2 g of leucine. Results showed significant increases in body weight, lean body mass, maximal extension, and flexion torque in both groups before and after supplementation, while muscle thickness tended to increase after 12 weeks of resistance. However, no significant differences were observed between the groups (Lynch et al., 2020). Another study also showed that habitual (over 12 months) vegetarians were given soy protein and omnivorous groups were given whey protein, with continuous supplementation at a protein intake of 1.6 g/kg/day combined with resistance training twice a week. After 12 weeks, strength, muscle mass, and cross-sectional area improved in both groups, but there were no differences in protein between the two groups (Hevia-Larraín et al., 2021). Although animal-based protein sources have long been considered to have higher absorption and utilization than plant-based protein sources, there appears to be little difference in muscle mass and functional performance. Therefore, vegetarians need more plant-based protein nutritional supplements to supplement their daily protein needs, especially elderly vegetarians. This study confirms that ISP supplementation in combination with resistance exercise training promotes and improves muscle mass and functional performance in the elderly and can serve as the basis for future applications and research in humans to help prevent and improve sarcopenia in elderly vegetarians.

## Conclusion

For the first time, we demonstrated that ISP supplementation in combination with RT effectively improved skeletal muscle mass, muscle endurance, and endurance performance of aging female mice. The RT group not only showed similar effects as ISP, but also increased muscle strength and glycogen content. Most importantly, the combination of ISP plus RT intervention in aging rats had greater beneficial effects than ISP and RT alone on various muscle strength and physical performance parameters. Therefore, age-induced muscle loss could be maintained and/or delayed through ISP supplementation and resistance exercise training strategies.

## Data Availability

The original contributions presented in the study are included in the article/supplementary material; further inquiries can be directed to the corresponding author.
